# Implications of current and future approaches to coronavirus disease 2019 testing

**DOI:** 10.2217/fvl-2020-0318

**Published:** 2020-10-13

**Authors:** Rasheid Smith, Sean M Geary, Aliasger K Salem

**Affiliations:** 1 Division of Pharmaceutics & Translational Therapeutics, College of Pharmacy, University of Iowa, Iowa City, IA 52242, USA

**Keywords:** coronavirus, COVID-19, diagnosis, immunoassay, lateral flow test, novel detection, RT-PCR, SARS-CoV-2, symptoms, testing

The relentless spread of severe acute respiratory syndrome-associated coronavirus-2 (SARS-CoV-2), the cause of the coronavirus disease 2019 (COVID-19) and the resulting pandemic has challenged the global economy, as well as impacting on the efficacy of governmental and medical systems to the serious detriment of not only those vulnerable to the potentially fatal consequences of becoming infected with SARS-CoV-2, but to the broader populace. Since the first reports of COVID-19 in Wuhan, China in December 2019, the scientific community has been in a race against time to elucidate the minutiae of the virus to inform public policy and implement appropriate strategies to maximize public safety. According to the weekly epidemiology report by the World Health Organization (WHO), as of 24 August 2020, 23 million people have contracted COVID-19 worldwide and this has led to 800,000 reported deaths though actual figures maybe higher due to inefficiencies in testing and reporting from individual nations. While SARS-CoV-2 has a case death rate in the USA of 3.1%, it can vary from as high as 28.9% (Yemen) to as low as 0.2% (Qatar) [1]. Socioeconomic factors greatly influence this case mortality as countries with resources and proper planning and implementation have reduced this to near zero in some cases. Analyzing the facts garnered to date about the SARS-CoV-2 virus, a major route of transmission is via droplets and aerosols emitted from infected individuals by coughing, sneezing or breathing [2]. Transmission through surfaces (fomite transmission) is assumed to be possible due to reports demonstrating that viable viruses can be found on surfaces such as plastic and stainless steel for up to 72 h after being administered with aerosols [3]. However, the applicability of this study in real life scenarios has been called into question due to high initial viral administration [4]. Nevertheless, transmission of SARS-CoV-2 via fomites has been listed as possible by WHO and the Centers for Disease Control and Prevention (CDC). SARS-CoV-2 has an incubation period of approximately 14 days with 97.5% of patients who will develop symptoms doing so within 11.5 days, on average [5]. There is an age-related disparity with respect to a patient’s susceptibility to infection with estimates that individuals over 20 years being approximately twice as likely to become infected as children (0–19 years) [6]. Additionally, SARS-CoV-2 infections in children/adolescents (ages 0–19 years) are often less severe [7,8], although cases of serious medical conditions such as hyperinflammatory shock have been documented [9]. Additionally, a significant portion of patients (adults/children) may present as asymptomatic (or subclinical presentation) [10–13], although the prevalence of this and how it relates to presymptomatic patients (patients who have not yet but will develop symptoms in a reasonable time) are not well understood. The complexities of COVID-19-related symptoms are still revealing themselves, as a growing body of research suggests that COVID-19 presentation is not limited to the respiratory system but symptoms may manifest as neurological (headaches, stroke) [14–16], gastrointestinal (diarrhea, nausea) [17,18] dermatological (erythematous rashes, widespread urticaria, chickenpox-like vesicles) [1,19], renal (proteinuria, hematuria), cardiovascular (myocardial ischemia, myocarditis) or endocrinal (diabetic ketoacidosis, hyperglycemia) [20]. This broad array of clinical presentations can be explained by the pathophysiology of SARS-CoV-2. The spike protein which facilitates cellular entry of SARS-CoV-2, binds to ACE-2 after priming by the host serine protease, TMPRSS2 [21]. ACE-2 is expressed by a broad array of tissues including cardiomyocytes, vasculature, gastrointestinal epithelia, and renal tubules [22] resulting in multiple potential viral entry ways. The presentation of multiorgan involvement is hypothesized to result from five mechanisms: direct viral toxicity, endothelial cell damage and thromboinflammation, dysregulation of the immune response and dysregulation of the renin–angiotensin–aldosterone system [20].

The current reality is that SARS-CoV-2 is a highly transmissible airborne disease with a broad presentation of symptoms and leaves lasting damage in severe cases, and for which there is a scarcity of effective medications to treat it. This coupled with the gaps in knowledge has resulted in a dire situation where implementing sound policy and planning has been the only reliable vanguard of public safety. Integral to ultimately ensuring public safety is the implementation of effective testing in order to: define what percentage of individuals are testing positive; identify trends in disease prevalence; identify potential epicenters of disease spread and facilitate swift measures to mitigate spread of the virus. The importance of testing has been aptly demonstrated by countries such as South Korea and Germany where increased availability of SARS-CoV-2 tests has resulted in a containment of viral outbreaks.

## COVID-19 testing & diagnosis

Under current US Food and Drug Administration (FDA) guidelines, COVID-19 testing is performed using an *in vitro* diagnostic device (a device used to diagnose a disease or condition from human samples). Such a device is granted emergency use authorization (EUA) in laboratories that are certified and meet guidelines governed under clinical laboratory improvement amendments of 1988 (CLIA) to perform high complexity (H), moderate complexity (M) testing or testing in a patient care setting (W) that has received an CLIA certificate of waiver. Testing thus can be performed in a centralized (i.e., samples are collected then transported to a facility certified to perform tests) or point of care (PoC; i.e., samples are tested immediately after sample collection often within the same vicinity of the patient) setting. Similar to other coronaviruses, the SARS-CoV-2 genome comprises positive sense single stranded RNA and is approximately 30 kB in length [23]. As such the gold standard for SARS-CoV-2 detection is through the reverse transcriptase polymerase chain reaction (RT-PCR). RT-PCR involves a two-step process converting RNA from samples (from nasopharyngeal swabs or saliva) to complimentary DNA and then using fluorescently labeled primers to amplify and detect the presence of targeted genes. The SARS-CoV-2 genes targeted for detection so far include: *RdRP*, *ORF1ab*, the *N* gene, the *E* gene and the *S* gene; with sensitivities ranging from 3.8 to 10 RNA copies present per reaction dependent on the gene used [24]. Currently, a large array of RT-PCR-based tests are available to detect COVID-19 as summarized in other publications [24,25] and there are currently 158 tests that have received EUA by the FDA [26]. Despite being the current gold standard, RT-PCR tests are not without their limitations, as many testing protocols are currently only using cycle threshold values (number of amplification cycles required for the signal to exceed the background levels) to determine infection status. Using the cycle threshold value in this manner only informs as to the presence of the virus and may not reveal disease progression, severity and viral load in the sample; and as such the results are largely qualitative despite the inherent quantitative nature of real-time RT-PCR [27]. Additionally, comparing the plethora of tests available is challenging due to inter-test variability due to specific viral genes targeted, primer design; differences in stated limit of detection (LOD) parameters (copies/ml, 50% tissue culture infective dose, copies/μl, copies per reaction volume). An additional limitation is the turnaround time for RT-PCR tests with average run times being approximately 4 h. In August 2020, the FDA gave EUA to two saliva-based tests (Saliva Direct, Yale University; I-COVID University of Illinois, Urbana Champaign, IL, USA) for SARS-CoV-2, which alleviates issues of the uncomfortable nature of the nasopharyngeal swabs [28]. Additionally, saliva-based tests limit the risk of infection to medical personnel as well as time required to collect samples as medical personnel are not required to collect samples. These saliva tests will provide the opportunity to lower the cost of collecting samples as they are generally easier to collect from patients.

Chest computed topography (CT) images have revealed characteristics common among COVID-19 patients including reverse-halo sign, consolidative opacities, ground glass opacities, interlobular septal thickening and the crazy-paving pattern [29–31]. As of the drafting of this manuscript chest CT images were not recommended by the CDC or the FDA for diagnosis of COVID-19 due to overlapping similarities with viral pneumonia, H1N1 and SARS CoV thus the possibility of false positive cases being reported with chest CT diagnosis is evident [24,32]. Nevertheless, initial studies have demonstrated that chest CT imaging is more accurate than RT-PCR at detecting SARS-CoV-2 patients [32] with 97.2% versus 83% in the early stages of infection [33]. Other drawbacks of implementing chest CT imaging as a mass diagnosis tool include processing costs and the technical skill required to accurately read chest CT images. Resultantly, chest CT imaging may be better used as a complimentary technology to molecular detection techniques such as RT-PCR and antibody serum tests.

Immunoassays (antibody serum tests), such as enzyme-linked immunosorbent assays (ELISAs), are used to detect the presence of serum antibodies (either IgA, IgG or IgM) to viral proteins and can indicate when a person has developed an immune response to SARS-CoV-2. The antibody response to any of SARS-CoV-2 four major structural proteins: spike (S) glycoprotein, small envelope (E) glycoprotein, membrane (M) glycoprotein and nucleocapsid (N) protein may vary with intensity and affinity during the course of an infection [34]. Infected individuals also go through antibody maturation and antibody class switching, refining the affinity and class, respectively, of antibodies produced to various epitopes, generally transitioning from (IgM to IgA or IgG) [35]. The sensitivity of immunoassays is dependent on the time of testing, viral antigen epitope used [36] and isotype of antibodies being detected [37]. The greatest sensitivity is observed when a mixture of antibodies is being detecting. Overall the ELISA technique is unsuitable for PoC testing due to the amount of labor and extended time it takes to generate results; however, in a centralized setting it may offer the advantages of determining antibody titers and selective isotype detection to monitor disease progression and the type/quality of immune responses [38]. Direct chemiluminescence immunoassays (dCLIs) may have advantages over ELISAs with run times of 1–2 h versus 4 h for ELISAs; and being automated, dCLIs are less labor intensive [38]. While COVID-19 serology tests are not used to diagnose COVID-19, the importance of serological testing in general is coming into focus currently with attempts to tackle unanswered questions about COVID-19. Questions such as the issue of post infection immunity and how it relates to antibody production and isotype switching in asymptomatic patients.

The aforementioned tests can generally be considered as centralized testing approaches where samples are collected and then results are generated by a certified laboratory that has expertise in consistently performing the test (e.g., chest CT, serology or RT-PCR). Under certain circumstances, such as during a pandemic, this system may be overwhelmed by the sheer volume of tests, resulting in significant lag times between testing and results [39]. Important tools that may alleviate the burden of these centralized approaches are PoC tests. PoC devices generally generate results more quickly and usually in a qualitative fashion. These technologies are based on a lateral flow assay, which is a paper-based platform for the detection of analytes (proteins and nucleic acids in complex mixtures), where the sample is placed on a test device and the results are displayed within 5–30 min [40]. However, issues as to the sensitivity of lateral flow immunoassays (LFIAs) have been raised when compared with centralized approaches such as dCLIs or ELISAs (e.g. 66% [LFIAs] vs 97% [dCLIs] vs 84.3% [ELISAs]) [41].

## Novel SARS-CoV-2 sensing

Notable advances in biosensing have allowed the rapid and accurate detection of SARS-CoV-2; the clusters of regularly interspaced short palindromic sequences (CRISPR)-based diagnostic product, Sherlock Biosciences’ 1-h test for SARS-CoV-2, is one such example. This test achieves detection of RNA (or DNA) virus signatures through two consecutive reactions: amplification of the viral RNA using an isothermal amplification reaction and detection of the resulting amplicon using CRISPR-mediated cleavage of the RNA based reporter (CRISPR-mediated collateral reporter unlocking) [42,43]. This test has also been validated to be 100% specific and 100% sensitive when using a fluorescence readout and 100% specific and 97% sensitive when using a lateral-flow readout [44]. Emerging technologies such as graphene field-effect transistors have been used to detect as low as 1 fg/ml of SARS CoV-2 spike protein in transport medium for nasopharyngeal swabs and detect SARS-CoV-2 in clinical samples with readings obtained within 1 min [45]. Additionally, Qui *et al.* used the plasmonic photothermal effect and a localized surface plasmon resonance sensing unit to attain real-time and label-free detection of SARS-CoV-2 viral sequences including *RdRp*, *ORF1ab* and *E* genes with an LOD of 0.22 ± 0.08 pM for the *RdRp* gene [46]. It is prudent to note that this study was performed in nuclease free water and therefore its compatibility with medical samples will still have to be evaluated. Silicon nanowire field-effect transistors have been previously used to provide label-free, real-time detection of proteins [47], DNA [48] and even single virions [49]; however, issues with sensitivity in physiologically relevant substrates and high manufacturing costs has hampered furthering this technology [50]. These issues have largely been solved in a variety of ways on a laboratory scale [51–53], thus employing these approaches toward SARS-CoV-2 detection (protein or genetic) is possible and could result in technologies that fill an important void in the current spectrum of testing methodologies.

Equally important to detecting positive cases is identifying the cases that will most likely transition to severe respiratory conditions and therefore requiring admission into intensive care units. This requires testing for additional biomarkers that adequately indicate disease severity and onset of symptoms such as acute inflammation. The Elecsys^®^ IL-6 immunoassay, which detects IL-6, an early marker from acute inflammation, is the only *in vitro* diagnostic test to have received EUA to manage COVID-19. Other cytokines and proteins can be implemented which are associated with increased severity of COVID-19 cases such as IL-2, IL-7, IL-10, G-CSF, IP-10 and cardiac troponin among others [13]. Park *et al.* have demonstrated that TGFβ-induced protein and its acetylated by-product are consistently elevated in the blood of SARS-CoV-2 pneumonia patients and tracks the severity of the SARS-CoV-2 infection [54].

## Conclusion

Ideally, a SARS-CoV-2 biosensor should provide accurate, sensitive, reproducible measurements of its target analyte in real-time and be cost-effective to the patient [50]. The present pandemic demonstrates the important impact that such effective biosensors could play in public health. However, the current iteration of tests seems to be divided into two camps: accurate/expensive methods of detection that have the potential for quantitative results but are associated with significant lag times and are only applicable in a centralized testing environment with trained personnel; or inexpensive PoC testing that offers qualitative results but may sacrifice sensitivity for ease of implementation and manufacturability. The current situation calls for the mobilization of both tactics to effectively help in dampening the spread of SARS-CoV-2. Qualitative tests are easy to produce en masse and accurately screen the population for SARS-CoV-2, thus helping to track the spread of the virus. Robust, quantitative tests are also required that offer results quickly to elucidate more information on the progression of SARS-CoV-2 and the physiological response to it. Advances in testing may therefore contribute to filling the current gaps in knowledge with regards to the SARS-CoV-2.
